# aMAP is a validated pipeline for registration and segmentation of high-resolution mouse brain data

**DOI:** 10.1038/ncomms11879

**Published:** 2016-07-07

**Authors:** Christian J. Niedworok, Alexander P. Y. Brown, M. Jorge Cardoso, Pavel Osten, Sebastien Ourselin, Marc Modat, Troy W. Margrie

**Affiliations:** 1 The Division of Neurophysiology, MRC National Institute for Medical Research, London NW7 1AA, UK; 2 The Sainsbury Wellcome Centre for Neural Circuits and Behaviour, University College London, London W1T 4JG, UK; 3 Translational Imaging Group, Centre for Medical Image Computing, University College London, London WC1E 6BT, UK; 4 Cold Spring Harbor Laboratory, Cold Spring Harbor, New York 11724, USA

## Abstract

The validation of automated image registration and segmentation is crucial for accurate and reliable mapping of brain connectivity and function in three-dimensional (3D) data sets. While validation standards are necessarily high and routinely met in the clinical arena, they have to date been lacking for high-resolution microscopy data sets obtained from the rodent brain. Here we present a tool for optimized automated mouse atlas propagation (aMAP) based on clinical registration software (NiftyReg) for anatomical segmentation of high-resolution 3D fluorescence images of the adult mouse brain. We empirically evaluate aMAP as a method for registration and subsequent segmentation by validating it against the performance of expert human raters. This study therefore establishes a benchmark standard for mapping the molecular function and cellular connectivity of the rodent brain.

## Abstract (web summary)

Anatomical segmentation of high-resolution 3D microscopy datasets is necessary to map large samples at cellular resolution. Here the authors present a pipeline for automated mouse atlas propagation (aMAP) to segment fluorescence images of the adult mouse brain and validate it against human segmentations.

## Graphical abstract

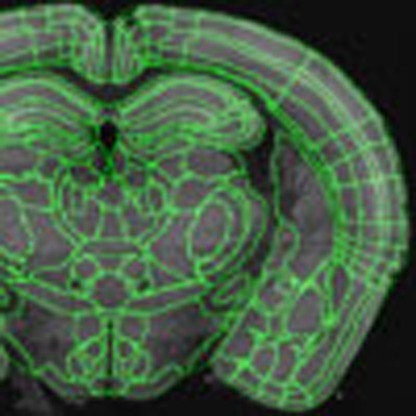

Brain-wide mapping of neuronal gene expression^1^, connectivity^2^,^3^,^4^ and function^5^ is required if we are to obtain a complete understanding of the physiological processes underlying cognition and behaviour. Recent advances in tissue clearing and high-resolution light microscopy^6^,^7^,^8^,^9^,^10^,^11^ combined with modern transgenic and neuronal tracing methods^12^,^13^ now make mapping of the mammalian brain with cellular resolution a feasible prospect^1^,^2^,^3^,^14^,^15^,^16^,^17^. However, any mapping effort requires the implementation of an objective, accurate and reliable means of defining the anatomical boundaries of underlying brain structures. The accuracy of this segmentation process is dependent on image registration and is critical since it defines the identity of cells or neuronal connections in terms of their anatomical position, a process that underpins interpretation and comparison across experiments.

Recently, automated high-resolution microscopy instruments have dramatically increased the throughput of data acquisition^8^,^11^ rendering manual segmentation an unfeasible prospect and necessitating the development of automated analytical pipelines. The most common approach for automating anatomical segmentation is called atlas propagation and involves performing registration of an image data set onto a standardized, fully segmented reference space to provide an anatomical segmentation of the original images^1^,^2^,^14^. One critical aspect regarding the implementation of such pipelines is ensuring that the quality of the resulting segmentation—previously achieved by expert neuroanatomists relying on their experience and detailed visual inspection of the data—is not compromised.

Such high-throughput microscopy instrumentation produces large volumes of high-resolution three-dimensional (3D) data and relies on the accuracy of automated segmentation, yet to date there has been only indirect assessment of segmentation quality and no agreement on a standard method of implementation, with individual labs using unpublished in-house tools^2^,^14^,^16^ or an open source clinical image registration tool (Elastix^18^) with unpublished parameters^1^,^17^. While these tools may perform adequately in their respective labs, only a validated, open source and fully automated method can enable the direct comparison of emerging data sets and cross-laboratory agreement.

Here we present aMAP, a tool that internally uses and provides a graphical front-end to NiftyReg (a rapid image registration toolkit, originally developed for human MRI data^19^), that we modified to enable rapid processing of high-resolution 3D light microscopy data. aMAP permits propagation of a 3D mouse atlas of the entire adult mouse brain in 40 min and its accuracy and reliability is shown to be on par with expert human raters.

## Results

### Assessing segmentation quality

**Figure 1:**
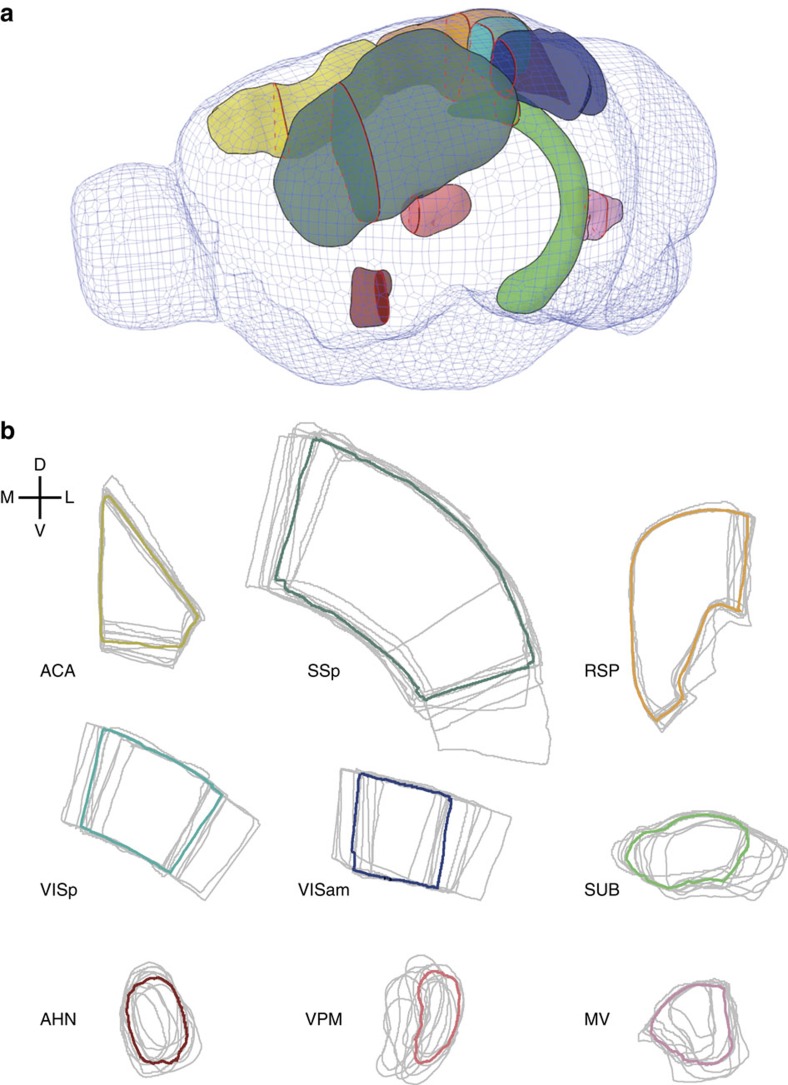
Anatomical structures used to assess segmentation performance. (**a**) An illustration showing the 3D shape of the nine brain structures in the left hemisphere used to assess segmentation performance. Red lines within each structure highlight the coronal plane in the reference atlas that was presented to human raters. (**b**) For each structure, the segmentation outlines are shown for a given group of 11 raters (grey lines). The consensus outline for the same structure and 11 raters as determined by STAPLE is overlaid (bold coloured line). According to The Allen Brain Atlas nomenclature, the nine structures shown are: anterior cingulate area (ACA); anterior hypothalamic nucleus (AHN); medial vestibular nucleus (MV); retrosplenial cortex (RSP); primary somatosensory area (SSp); subiculum (SUB); primary visual cortex (VISp), secondary visual cortex, anteriomedial part (VISam); ventral posteromedial nucleus of the thalamus (VPM) (D: dorsal; V: ventral; M: medial; L: lateral).

To assess the performance of human raters on manual segmentation, twenty-two neuroscientists were randomly assigned to one of two groups and asked to segment the same ten target structures (of which 9 were analysed; see Methods; Fig. 1a) from three brain datasets. Target structures were presented within six serial two-photon (STP) image stacks (40 coronal planes per stack containing tissue background fluorescence (*n*=5 brains) or sparse red fluorescent protein (RFP) labelling (*n*=1 brain)) obtained from adult C57BL/6 mice. These structures were chosen to encompass a broad range of sizes and anticipated difficulty based on their degree of border definition according to local anatomical landmarks. Raters were required to identify one image plane from the STP stack that best matched the target section presented from the two-dimensional (2D) anatomical reference atlas of the Allen Brain Institute^14^ and then asked to manually outline the perimeter of the target structure on the STP image (see Methods).

Qualitatively, human raters showed substantial inter-rater variability in their positioning of the borders and estimation of the size of target structures (Fig. 1b). In general, there was stronger agreement—that is low inter-rater variability—where the structure could be identified using high-contrast landmarks, such as structure borders at the ventral and dorsal surfaces of the neocortex. However, we observed weak agreement (high inter-rater variability) at borders that were less well delineated, such as the cortico-cortical boundaries of cortical target regions (for example, primary visual cortex (VISp), Fig. 1b). Disagreement between raters was particularly significant for target structures that lacked any distinct anatomical landmarks, such as the ventral posteromedial nucleus of the thalamus (VPM), the segmentations of which, showed very little overlap in boundary definition (Fig. 1b).

Recent approaches to validating mouse-brain segmentation have relied on comparing the Euclidean distance between manually chosen anatomical landmarks in an image data set before and after its registration (step 1; Supplementary Fig. 1) to an average brain image^1^,^9^. While this method is easily implemented, it can only report registration accuracy proximal to the chosen landmarks and is not indicative of the quality of segmentation (step 2; Supplementary Fig. 1).

On the other hand, direct assessment of segmentation quality is hindered by the fact that there is no ‘ground truth' regarding the precise location of an anatomical structure in any data set. Thus, it is not possible to assess the quality of either automated or human segmentations without first establishing a ‘ground truth' segmentation of the image data sets. To achieve this essential initial step we therefore determined the consensus segmentation of all human raters for each target structure in each brain data set using STAPLE^20^, an iterative algorithm that—when given multiple segmentations—simultaneously estimates the quality of each segmentation and derives the quality-weighted consensus (see Methods). Using the STAPLE consensus segmentation as a ‘ground truth' we could now directly evaluate segmentation performance of both human raters and aMAP using the Dice score metric^21^ that quantifies the overlap between two structures and is commonly used to assess automated segmentation quality^22^.

Consistent with the idea that the STAPLE–Dice method is directly reporting the quality of segmentation, we first determined that imposing a goodness of fit on the registration of STP images to an average brain data set^1^,^9^ (that is, by constraining the bending energy) exerted a significant influence on Dice scores that improved with increasing bending energy weight (repeated measures ANOVA, *F*_(15,75)_=16.8, *P*<0,001; Supplementary Fig. 2a (range 0.2–0.95)). In contrast, the Euclidian distance between landmarks was insensitive to changes in the goodness of fit of the registration imposed by the same range of bending energy weight (repeated ANOVA, *F*_(15,75)_=0.45, *P*=0.95 Supplementary Fig. 2b).

To score segmentation quality of both human raters and aMAP, we next compared each segmentation with the appropriate STAPLE consensus using the Dice score. As a complimentary measure, we also used shape-based averaging (SBA)^23^ to generate an average segmentation of human raters and the Hausdorff metric as a second segmentation quality metric (see Methods, Supplementary Fig. 3a,b). Although these different methods for determining the ground truth segmentation and segmentation quality produced very similar results (Supplementary Fig. 3a,b), we adopted the STAPLE–Dice metric, as it is the most widely accepted analytical tool used in other imaging fields^22^.

aMAP was implemented using the open-source NiftyReg toolkit^19^ to register the average brain of the Kim *et al*.^1^ 3D atlas to downsampled versions of our STP data sets (12.5 μm isotropic) using affine and free-form registration. The resulting transformations were then applied to the 3D Kim *et al*.^1^ brain atlas, which is based on the Allen Institute Brain Atlas that was used here by human raters. Computation time for aMAP-based segmentation was 40 min per downscaled brain on a dual-6-core Xeon workstation. To find the appropriate parameters for the image registration, we used two of the six STP brains as training sets. Unless noted, these brains were excluded from the analysis of aMAP's performance.

### Human raters versus aMAP

**Figure 2:**
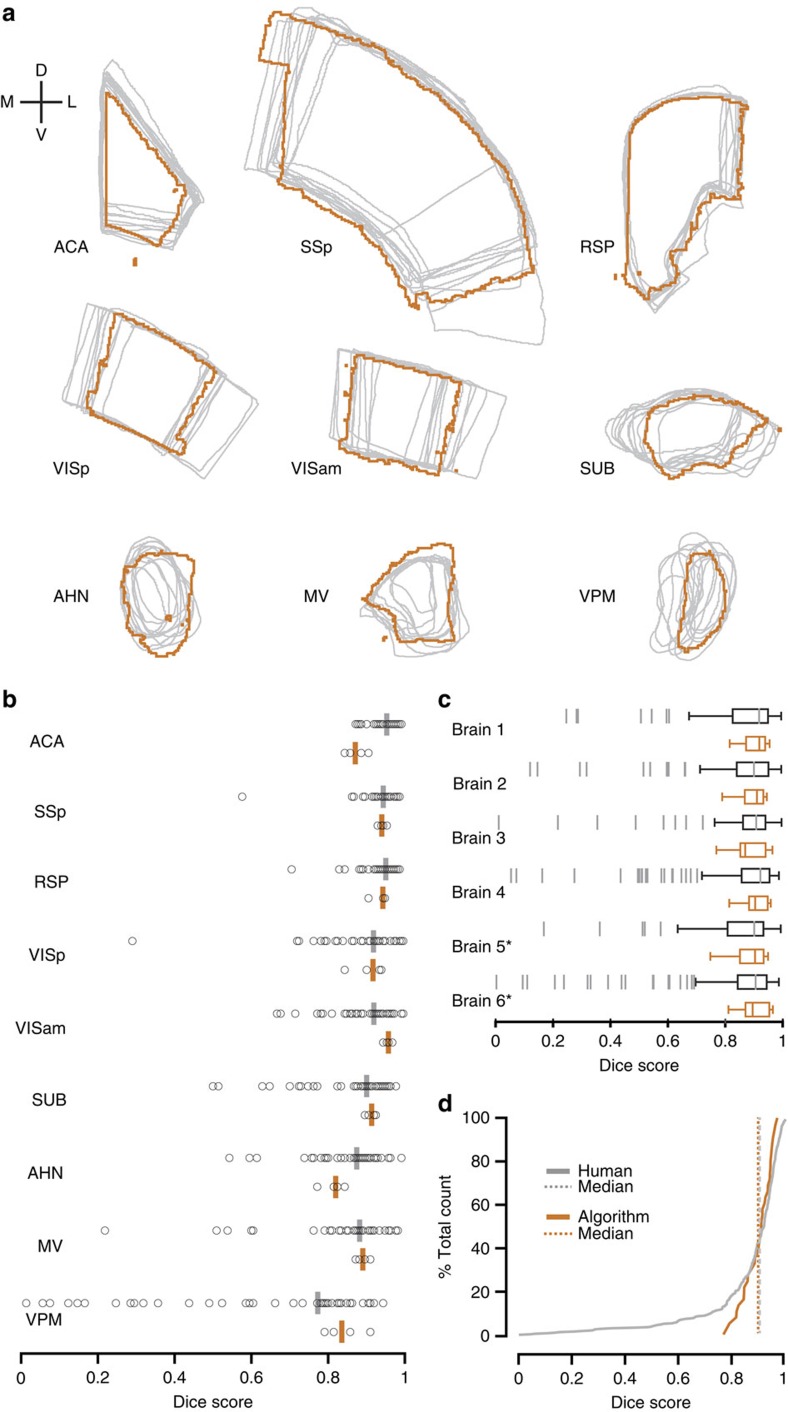
Segmentation performance of human raters and aMAP. (**a**) Segmentation outlines of human raters (grey) with the aMAP segmentation result of the same structure and brain overlaid (orange). (**b**) Dice scores for manual (*n*=22 raters, each segmenting two of four potential brains, grey) versus aMAP (*n*=4 brains, orange) segmentations grouped by target structures (*n*=9). (**c**) Box plots showing Dice scores of human (grey) versus aMAP (orange) segmentations grouped by brain. Brains used in the registration parameter search (training data) are marked with an asterisk. (**d**) Cumulative histogram of the Dice scores for manual (grey) and aMAP (orange) segmentations for all structures and brains as shown in **b**. Vertical lines indicate the median scores.

The outlines obtained from aMAP (Fig. 2a, orange lines) were qualitatively similar to those performed by human raters (Fig. 2a, grey lines), which was confirmed by Dice score analysis (Fig. 2b–d). When pooling the scores of all structures, the median score achieved by aMAP was not significantly different from human performance levels (Mann–Whitney *U*-test, score of 0.92 versus 0.91, *P*=0.52; *n*=4 brains, 9 structures, 22 human raters). When grouping these scores by structure, there were no significant differences between the scores for human raters and aMAP in eight out of nine structures. Humans scored significantly better in segmenting the anterior cingulate area (ACA, Mann–Whitney *U*-test, median Dice score of 0.952 versus 0.870, *P*=0.005; Fig. 2b). When grouping the scores by brain rather than structure, there were no significant differences observed between human raters and aMAP for any individual brain (Mann–Whitney *U*-test, *P*>0.49, Fig. 2c).

**Figure 3:**
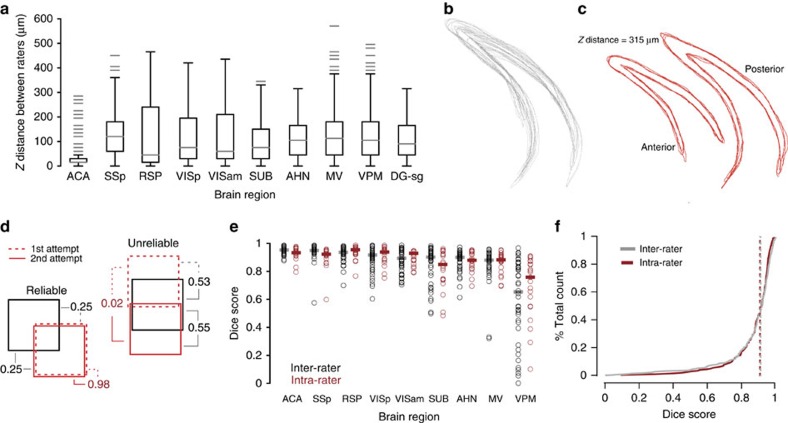
Sources of variance in manual segmentation. (**a**) A box plot showing the anterior–posterior distance between any two human raters (*n*=22 raters, each segmenting three of six potential brains) in their estimation of the correct optical section (*z*-choice) for manual segmentation for each brain structure. Structures: anterior cingulate area (ACA); anterior hypthalamic nucleus (AHN); dentate gyrus, granule cell layer (DG-sg); medial vestibular nucleus (MV); retrosplenial cortex (RSP); primary somatosensory area (SSp); subiculum (SUB); primary visual cortex (VISp); secondary visual cortex, anteriomedial part (VISam); ventral posteromedial nucleus of the thalamus (VPM). (**b**) Example manual segmentations (*n*=22) of the DG-sg performed by 11 human raters taken from a single test brain and its repeated presentation. (**c**) Example manual segmentations of the DG-sg, taken from two *z*-sections from within the data set shown in **b**. These two *z*-sections were chosen based on their having multiple segmentation attempts (*n*=4 outlines shown in each image, left image: anterior, right image: posterior). (**d**) Schematic highlighting two extreme segmentation reliability scenarios. Bottom left: a given rater may perform poorly against the STAPLE consensus (black square) of all raters (Dice score=0.25, grey lines). However, generation of a Dice score that determines the overlap between the first attempt and the second attempt (intra-rater Dice score, red) indicates high reliability (for example, Dice score=0.98). In contrast, a given rater may obtain a Dice score more similar to the STAPLE consensus but be unreliable in their estimate of the location of the structure (for example, intra-rater Dice score=0.02; top right). (**e**) Plot of the inter-rater (*n*=44 segmentations per structure; that is, first and second attempt versus STAPLE consensus for 22 raters per structure, black) and intra-rater (*n*=22 segmentations, that is, second attempt versus first attempt for 22 raters per structure) Dice scores for each target structure. (**f**) Plot showing the cumulative histogram of intra- and inter-rater Dice scores for all data presented in **e**.

However, despite there being no significant difference in the overall median scores between human raters and aMAP, human raters exhibited substantial variance, while the Dice scores obtained by aMAP were significantly more consistent (Levene's test on pooled scores; *n*=4 brains, 9 structures, 22 human raters; s.d.: 0.16 versus 0.05, *P*=0.005, Fig. 2d). In addition to the variance observed for *x*–*y* border definitions, human raters strongly disagreed as to which optical section of the STP data sets best corresponded to the Allen Brain Atlas plates, leading to substantial variation in the identity of the section chosen for segmentation (*z*-choice, Fig. 3a; median anterior–posterior distance between two raters on the same brain and structure (*n*=6 brains)—ACA: 15 μm, primary somatosensory area (SSp): 120 μm, retrosplenial cortex (RSP): 45 μm, VISp: 75 μm, secondary visual cortex, anteriomedial part (VISam): 60 μm, subiculum (SUB): 75 μm, anterior hypothalamic nucleus (AHN): 105 μm, medial vestibular nucleus (MV): 112.5 μm, ventral posteromedial nucleus of the thalamus (VPM): 105 μm, dentate gyrus, granular cell layer (DG-sg): 90 μm). Such differences in *z*-choice had a particularly strong influence on segmentations of DG-sg that resulted in substantial discrepancies in *x*–*y* border definitions (Fig. 3b), despite the fact that the structure could be clearly delineated in the STP data set (Supplementary Fig. 4). Thus while manual segmentations performed on the same optical plane of the dentate gyrus generally showed good agreement (Fig. 3c), the overall *x*–*y* boundary of the DG-sg changed substantially according to the choice of *z*-section (Supplementary Fig. 4). We therefore excluded this structure from the segmentation analysis, since the discrepancies in segmentations were substantially negatively influenced by differences in *z*-choice rather than a rater's uncertainty about the *x*–*y* boundary of the structure. We found no significant influence of the *z*-choice range on the median Dice scores of human raters for the remaining structures (*P*>0.05, Supplementary Fig. 5).

### Intra-rater reliability

By design, aMAP will always produce an identical segmentation when applied to the same data set. In contrast, one major source of the significant inter-rater disagreement on manual segmentations could be a rater's degree of reliability. Although an individual may score poorly compared with the STAPLE consensus, they may nevertheless be extremely reliable in their estimate of the location and shape of the target structure (Fig. 3d). On the other hand, the trial-to-trial reliability of a rater could significantly contribute to the broad range of (inter-rater) Dice scores. Reliability could therefore be considered to be one major source of variability inherent in the segmentation process and distinct from inter-rater disagreement.

To investigate the extent to which inter-rater disagreement in the segmentation stemmed from an individual's uncertainty or from a reliable difference in opinion between raters, each user was unknowingly also presented with repeats of all target structures from one of their three previously presented brains. This permitted calculation of a Dice score between a rater's first segmentation and the repeat segmentation of the same structure in the same data set (intra-rater Dice, Fig. 3d). Comparison of the intra-rater Dice score with the previously determined inter-rater scores from the same brains and structures (Fig. 3d) showed significantly worse intra-rater performance on the ACA and SSp, (Mann–Whitney *U*-test, inter versus intra: ACA: median 0.953 versus 0.933, *P*=0.01; SSp: median 0.950 versus 0.923, *P*=0.001; Fig. 3e, *n*=2 brains × 9 structures × 11 raters per brain) and no significant difference on the remaining structures (Mann–Whitney *U*-test, *P*>0.21). There was no significant difference in the overall median of inter- versus intra-rater scores (Mann–Whitney *U*-test, inter versus intra: 0.916 versus 0.912; *P*=0.32) and only a modest but significant reduction in variance (Levene's Test, inter versus intra: s.d.; 0.16 versus 0.12, *P*=0.044, Fig. 3f). This indicates that for a given rater, there exists substantial variability in repeated segmentation of the same structure. Thus, human inconsistency is a significant source underlying segmentation disagreement between individual raters.

## Discussion

Any attempt to map the brain with cellular resolution depends critically on an objective, accurate and reliable means of defining its underlying architecture. In this study, we have directly compared the performance of an algorithm for automated segmentation of high-resolution 3D fluorescence data sets with the segmentation performance of human raters. We show that manual segmentation is a process that, on average, is of high quality but with modest reliability. While human raters—as a group—generally achieved high median scores, they displayed significant variability, particularly for structures that did not follow obvious anatomical landmarks. The fact that this variability was at least as large for intra-rater comparisons as it was between raters highlights that both the accuracy and reproducibility of manual segmentation is inherently limited. On the other hand, aMAP performed just as accurately as human raters, but with significantly less variability and, by design, is entirely reproducible.

We have designed aMAP based on NiftyReg because of the high speed of its free-form registration and the possibility to adapt it to large data sets. There are however several other applicable MRI registration tools in use in the clinical field^24^ that may be equally suitable for 3D fluorescence data. Previous rodent brain microscopy studies have used pipelines based on such tools (for example Elastix^1^,^17^ and MNI AutoReg^16^) or unpublished in-house tools^2^,^14^, but did either not publish validation data of their image analysis pipeline^16^,^17^, or validated their segmentation by relying on a landmark distance-based measure that determines the Euclidian distance between a limited number of point markers in the registered data set^1^,^2^,^14^. Here we show that the landmark distance metric does not capture changes in registration quality over a wide range of deformations imposed on the image data set that, by its very nature, impacts the quality of the segmentation process. It is worth noting, that results from previous studies relying on the distance between point markers may nevertheless be accurate. However, our data mirrors previous findings showing that such scoring metrics do not capture the quality of free-form registration of MRI data sets^25^. To encourage community-wide implementation and validation of automated segmentation tools, we have made our manual segmentation data and validation pipeline freely available (see Methods).

In our study, we have ensured parallelity of the optical sections to the coronal plane of the atlas by rigidly registering all images to the Allen average brain. Furthermore, we specified to the human rater the atlas sections that contain the target structure. Nevertheless, differences in the *z*-plane chosen by the raters from the optical stack could remain a significant source of inter-rater variability in segmentation performance. However, at least for the structures analysed here, we found that Dice scores were not significantly improved when our segmentation analysis was confined to optical planes within seven or three sections of one another. It is of course also conceivable that in the real-world scenario both image misalignment and lack of agreement on the correct atlas section could further increase inter-rater variability.

Agreement can be achieved by using several experts to cross-validate segmentations, a practice that is widely used on MRI data in the clinic^20^. However, since high-resolution whole-brain fluorescence data sets are typically several orders of magnitude larger than MRI data sets, this approach is extremely difficult to implement without substantially down-sampling and thereby compromising accuracy. Also, manually agreeing on brain-wide segmentation of high-resolution images is a very laborious and time-consuming process. Particularly for high-throughput pipelines, validation using a limited number of agreed expert raters is impractical and would slow what is already a major analytical bottleneck.

The success of registration depends on the similarity of the images being registered to one another. As such, the location and integrity of key anatomical landmarks (such as the cortical surface) are critical to accurate brain registration. To maximize similarity between our data and the atlas average brain data set (which was generated using tissue autofluorescence) we have used either the background fluorescence or a sparsely labelled RFP channel. In contrast to using fluorescence images exhibiting for example, a very specific anatomical pattern of GFP, this ensures that most pixel values in the image reflect anatomical structures. While aMAP can theoretically be used on image data containing fluorescent signals, it is not possible to reliably predict the impact of such signal patterns on the registration process. We therefore recommend manual quality assessment of the images and their segmentation, especially in cases where specimens have suffered dissection-related damage or that contain excessive imaging artefacts, such as high non-specific background fluorescence (e.g. due to a failed perfusion). We found that overlaying the original image data with the registered average brain and the segmentation outlines provides a reasonable way to qualitatively assess image registration and segmentation.

One shortcoming of all current atlas-based automated segmentation approaches arises from the fact that existing 3D atlases either have (i) adequate 3D segmentation but contain a limited number of annotated structures, as is the case for mouse MRI atlases^26^,^27^,^28^ or (ii) have a reasonable number of annotated structures but are based on reconstructions of serial 2D sections rather than genuine 3D segmentations^1^,^14^. This latter scenario unfortunately leads to discontinuity in structure borders in the plane orthogonal to the atlas's cutting plane^29^ that will propagate into any automated segmentation based on such an atlas (Fig. 2a; Supplementary Fig. 6). Despite this limitation, aMAP nevertheless performs on par with human raters and its implementation provides a means of establishing an agreed standard for automated segmentation. Fortunately, in its most recent release, the Allen Brain Institute has begun to move towards a higher resolution 3D-segmented atlas. Although this recent version contains a mixture of 2D and 3D annotations, the goal is to eventually generate an atlas that is fully annotated in 3D. This represents a crucial step forward that will further improve the quality of automated segmentation.

A recent development in the field of MRI imaging has been the introduction of multi-atlas registration to increase the robustness of automated segmentation^30^,^31^,^32^,^33^. There, multiple atlases are registered to the data set of interest and the final segmentation is generated using the consensus segmentations from all individual atlases. While this method increases the robustness of the automated segmentation, the high-resolution multi-atlas datasets necessary to implement this method on 3D light microscopy data do not yet exist.

The fact that automated segmentation will, by design, adapt to future atlas releases highlights another important aspect: Automated segmentation generally works by mapping all points of interest in the experimental data (for example, neuronal and glial somata) to a common reference space. As refinements are made in the segmentation of this common space or new areas are functionally delineated, tools such as aMAP can be used to systematically apply these changes to existing and previously published data sets (assuming points of interest are published using reference space coordinates). In this way, pipelines such as aMAP will enable the data of previous and future studies to be directly compared as 3D mouse atlases evolve.

In summary, we have for the first time validated a tool for segmenting high-resolution 3D imaging data that will rapidly register and segment a complete adult mouse brain data set. aMAP performs as well as human raters but with substantially less variability and thus enables direct comparison of anatomical data sets independent of the level of experience and knowledge base of the user. aMAP can therefore be used to standardize the segmentation process and enable comparability of data from one individual and lab to another. Furthermore aMAP will, by design, inherently adapt to any future refinements in digital segmentation atlases, the precise application of which is currently a significant factor limiting the accuracy of brain-wide mapping approaches.

## Methods

### Imaging

Male adult C57BL/6 mice were trans-cardially perfused with cold 4% PFA-solution under general anaesthesia. Brains were then removed and post-fixed in 4% PFA for at least 24 h. All procedures were in accordance with UK Home Office regulations (Animal Welfare Act 2006) and the local animal ethics committee. Brains were imaged coronally at a voxel size of 1 μm (*x*) × 1 μm (*y*) × 5 μm (*z*) under a STP microscope^9^ using an Olympus × 10 water immersion objective (numerical aperture 0.6). The STP image files for all target brains were rigidly aligned to the 3D average brain of the Allen Mouse Brain Atlas^14^ to ensure optical sectioning in the coronal plane of all datasets. The transformation matrices were determined on *z*-smoothed (Gaussian, 5 voxel s.d.) and then downscaled versions of background fluorescence (*n*=5) or sparsely labelled RFP (*n*=1) images using NiftyReg (reg_aladin^34^, voxel size 12.5 μm isotropic, https://sourceforge.net/projects/niftyreg). The resulting transformation matrices were then applied to the full-resolution images using MATLAB (MathWorks).

### Segmentation task

Manual segmentation data was obtained from a group of 22 neuroscientists that included postgraduate students, research assistants, postdoctoral fellows and principal investigators. This cohort was randomly split into two groups (*n*=11 raters per group) whereby every rater from within a group performed 10 segmentations on each of three brains (three different brains per group). In addition, all 10 structures from one of the three brains were re-presented blindly as a fourth data set to assess intra-rater reliability. For inter-rater analyses, only the first segmentation of the repeated brain was used.

Raters were asked to segment the following structures on one hemisphere of the brain: ACA; AHN; MV; RSP; SSp; SUB; VISp; VISam VPM and DG-sg. During the analysis, we found strong influence of the z-choice on the human DG-sg segmentations. We therefore excluded this structure from segmentation analysis (see Results). For each target structure, the task proceeded as follows: an STP stack consisting of 40 images (step size: 15 μm) was presented to the rater on a digitizer-pen-enabled monitor (Wacom Cintiq 22HD). The rater was also presented with a single plate from the online version of the Allen Mouse Brain Atlas on a second monitor and asked to outline the target structure in the STP data set. The raters were free to browse all Allen atlas plates to orient themselves along the anterior–posterior axis if necessary. The image stacks were presented using a custom Fiji/ImageJ^35^,^36^ plugin that handled loading of images and logging of results. The order in which the brain structures were presented was random but identical for each participant within a group. Each set (*n*=4) of 10 different structures was sampled from multiple brains.

### Scoring and analysis

Manual segmentations were first manually cleaned by removing, for example, isolated touches that may appear when a rater accidentally clicks on an unrelated part of the data set in draw mode. From a total of 880 segmentations, we found five cases where the rater segmented the wrong structure or hemisphere. These cases were not included in the analyses. The remaining outlines of the segmented target structures were converted to filled binary images and downscaled to a pixel size of 4 μm in *x*–*y*. Due to the lack of a ground truth, all segmentations for a given target structure were compared to an ‘consensus segmentation' derived from all manual segmentations of that structure using STAPLE. STAPLE is an iterative algorithm, designed to simultaneously assess the ‘quality' of each segmentation and the average of all segmentations weighted by their quality. Quality is derived from the overlap of each segmentation with the agreement structure and is initialized to equal levels for all segmentations^20^.

As an additional measure, we also calculated the inter-rater agreement using SBA^23^, which gives the geometric mean of all segmentations. Both averaging methods yielded similar results (Supplementary Fig. 3a,b). Both the STAPLE and SBA consensus structure for each target were calculated using NiftySeg (seg_maths^37^, https://sourceforge.net/projects/niftyseg). To score the quality of manual and automated segmentations, individual segmentations were compared to the consensus segmentation using the Dice score^21^. The Dice score is generally defined as the area of the intersection of two sets (that is, segmentations) divided by half the sum of the sets' areas and thus provides a measure of relative overlap between two segmentations. The Hausdorff metric was used as a supplementary scoring method (Supplementary Fig. 3b) and is generally defined as the longest distance between any point on one set and its closest neighbour in the other set and thus provides a good measure for the maximum distance between two segmentations.

Non-parametric tests were used to determine statistical significance, since data were found to be not normally distributed. Based on the observed effect sizes and number of repeats used for both manual and automated segmentations, such tests were performed with 100% power when the confidence interval was set to 99%.

### Automated segmentation

The average brain data set from 3D mouse brain atlas by Kim *et al*. was aligned to the *z*-smoothed (Gaussian, 5 voxel s.d.) and then downsampled (12.5 μm per voxel isotropic) versions of the six brain data sets that had been used for manual segmentation. For registration, we used either the background fluorescence channel (*n*=5) or a sparsely labelled RFP staining (*n*=1). The first alignment step was an affine registration (NiftyReg, reg_aladin^34^, six levels coarse-to-fine pyramidal approach of which the first five steps were computed) using a symmetric block-matching approach^34^.

This was followed by a second free-form registration step, which places a regular grid of control points onto the reference image (NiftyReg, reg_f3d^19^). These control points are moved during registration, causing the surrounding image data to be moved, allowing for a local, non-linear alignment of the image data^38^. A parameter search was performed on two of the six brains to find suitable parameters for the free-form registration. Since image registration is a step-wise process that relies on assessing a cost function that embeds a measure of similarity between two data sets, we tested two similarity measures that both compare relative intensity differences in the atlas and the brain to be segmented: locally normalized cross-correlation and normalized mutual information^19^. Normalized mutual information, using 128 bins discretization achieved the highest overlap score and was hence used for aMAP. The remaining parameters achieving the highest overlap score were an initial Gaussian smoothing of the input images (with a 1 voxel s.d.), a control point grid spacing of 10 voxels isotropic, a bending energy weight of 0.95 and a six levels coarse-to-fine pyramidal approach of which only the first four steps were computed. For a more detailed description of the parameters, see the software manual distributed with aMAP. Unless specifically noted, the two brains on which the parameter search was performed were excluded from the analytical comparison of the manual versus automated segmentation.

The transformations obtained from registering the Kim *et al*. average brain to the individual image data sets were then applied to the 3D atlas from Kim *et al*. (Supplementary Fig. 1). Since this atlas is based on the original Allen brain atlas (generated from individual 2D segmentations of nissl-stained coronal plates^29^), structures show high-frequency fluctuations in border definition along the axis orthogonal to the atlas plane of section (Supplementary Fig. 6). To minimize their impact, the 3D atlas was smoothed twice using a Gaussian kernel with a s.d. of 0.5 voxels prior to being transformed (NiftySeg, seg_maths). Since the aMAP segmentations are 3D volumes, they cannot be directly compared with the 2D segmentations of human raters. Hence, the 3D volumes were converted to 2D outlines by making coronal sections through the part of the aMAP-generated 3D segmentation that corresponded to the stack given to human raters. The 2D outline with the highest Dice score was chosen as the result of the automated segmentation.

### *z*-distance scoring

To find the median distance between the sections chosen from the 40 optical sections in the STP data sets, each rater's *z*-choice was first determined and compared with the *z*-choice of all other raters segmenting the same STP data set. The absolute difference in section number for any two raters was then converted to distance by multiplying with the *z*-distance between two optical sections (15 μm). We calculated the absolute distance in *z* between two raters on the same brain and structure for all possible non-ordered combinations of raters.

### Comparison of Dice- and landmark distance scoring

The following anatomical landmarks, as defined by the Waxholm space^27^, (http://scalablebrainatlas.incf.org/main/coronal3d.php?template=WHS11&) and used in ref. 1, were placed in the average brain of the Kim *et al*. atlas and the downscaled version of each STP brain before registration: frontal middle 1; frontal right 2; frontal left 2; anterior commissure right; anterior commissure left; corpus callosum middle; hippocampus middle; interpeduncular nucleus right; interpeduncular nucleus middle; interpeduncular nucleus left. In one STP brain, hippocampus middle was omitted due to an imaging artefact in that region. The free-form registration was then rerun for each of the 6 brains using 18 different imposed bending energy (BE) weights (range: 0.2–0.99). The BE is used to penalize high-frequency transformation and acts as a regularization term in the optimization process. The optimization aims to find the best transformation parameters by maximizing the image similarity while minimizing the transformation BE. A BE weight that is set too low will lead to a mismatch of the segmentation due to artefacts caused by over-fitting the images. Setting the BE weight too high, on the other hand, will overly constrain the registration resulting in a more global mismatch between the segmentation outlines and the target brain. For the analysis of the suitability of the Dice score metric, the mean Dice score of all target structures in each brain (10 structures per brain) were plotted against the BE weight. Likewise, for the landmark distance analysis the mean distance between the landmarks in the brain data sets and the registered atlas were plotted against the BE weight.

### Influence of *z*-range on Dice scores of human raters

To test whether the range of *z*-sections chosen by the human raters had a significant influence on the Dice scores of raters, we reanalysed a subset of the manual segmentations, choosing a window of three and seven consecutive *z*-sections for each structure in each brain. All segmentations that were not performed in this window were then discarded for this analysis. The position of the window was chosen on each brain and structure to contain the maximum possible number of human segmentations. New STAPLE consensus segmentations were generated for the *z*-limited analysis.

### Brain structure schematic

To illustrate the position of the analysed structures and sections, 3D models were generated from the Allen Mouse Brain 3D voxel data using Fiji/ImageJ^35^,^36^ to generate mesh models and blender (www.blender.org) to remesh, smooth and render them.

### Data availability

Detailed instructions for setting up and using aMAP, including all necessary data and software, are openly available at http://www.swc.ucl.ac.uk/aMAP. This url also provides the published manual segmentations and validation pipeline and instructions on how to adapt the validation pipeline for other segmentation software.

## Additional information

**How to cite this article:** Niedworok, C. J. *et al*. aMAP is a validated pipeline for registration and segmentation of high-resolution mouse brain data. *Nat. Commun.* 7:11879 doi: 10.1038/ncomms11879 (2016).

## Supplementary Material

Supplementary Information Supplementary Figures 1-6
