# Supplementary material for: aMAP is a validated pipeline for registration and segmentation of high-resolution mouse brain data
Source: Nat Commun. 2016 Jul 7;7:11879. doi: 10.1038/ncomms11879 (PMC4941048; doi:10.1038/ncomms11879)
Supplement: Supplementary Information — Supplementary Figures 1-6 [file ncomms11879-s1.pdf]

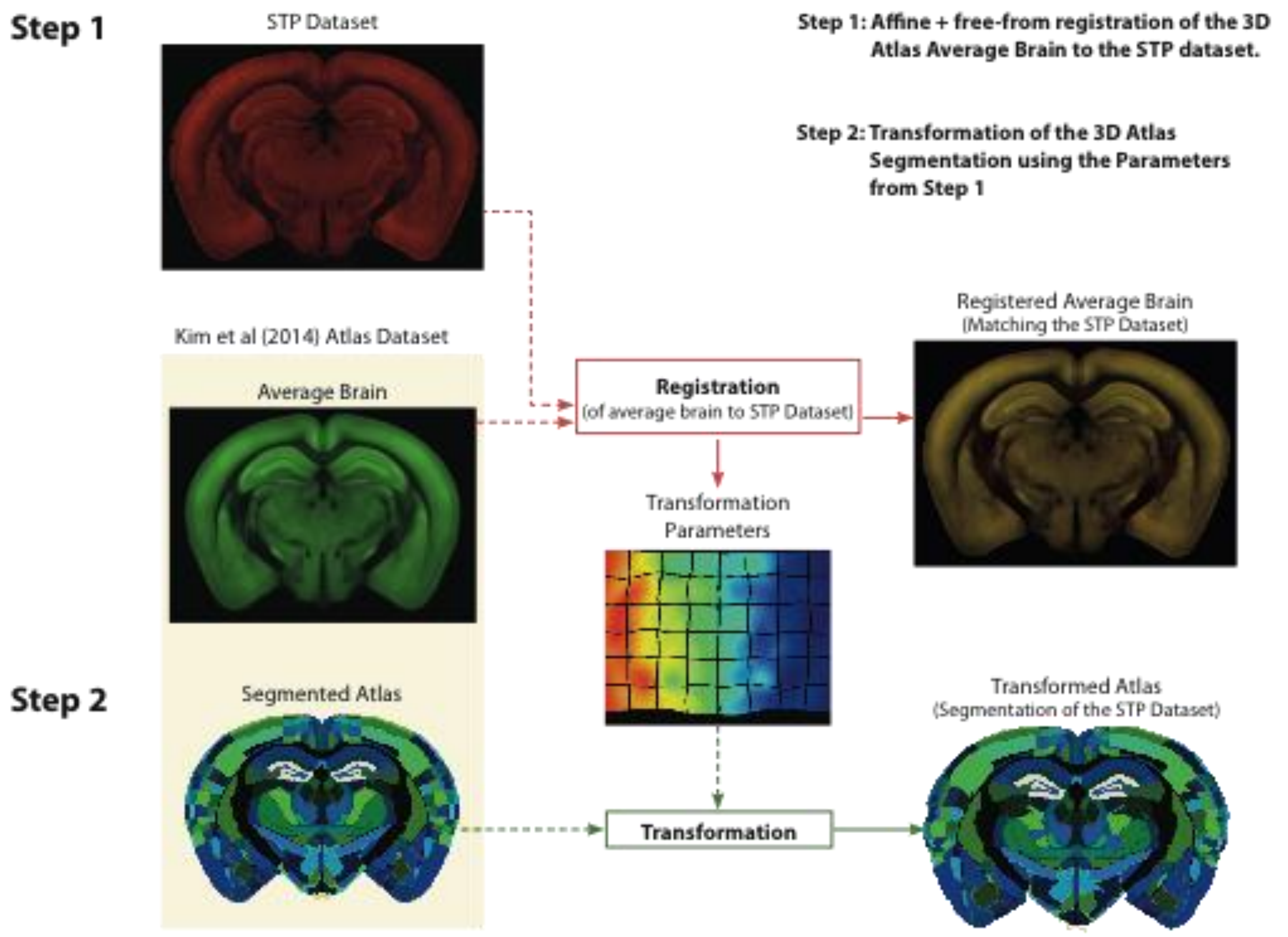

Supplementary Figure 1: Schematic of the aMAP segmentation propagation. In a first step, the average brain dataset of the Kim et al. 3D mouse atlas is registered to an individual 3D STP dataset, using both, affine and free-form registration. As a result, the registered average brain now matches the shape of the STP dataset. The transformation parameters that describe the image registration are then applied to the 3D segmentation file that contains the brain structure outlines of the mouse atlas. As a result, the transformed segmentation file now contains the brain structure outlines describing the anatomy of the STP dataset.

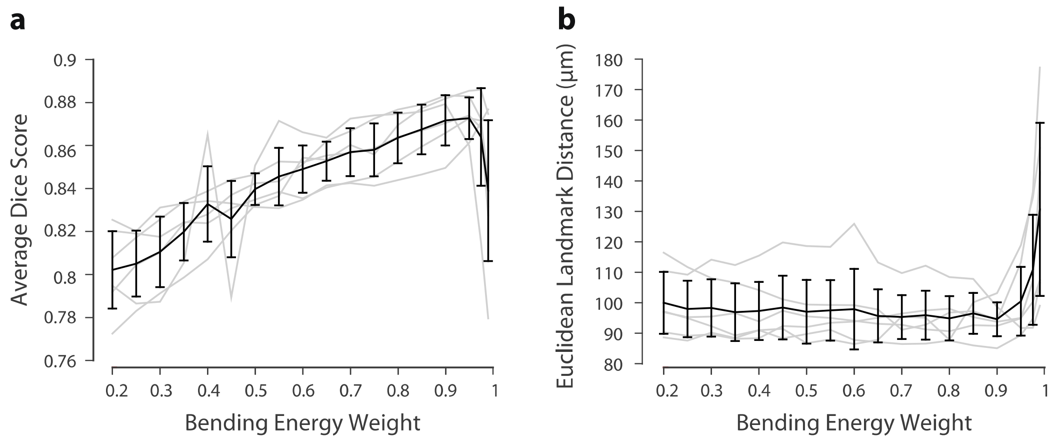

Supplementary Figure 2: Sensitivity of Staple-Dice and Landmark Distance scoring to changes in registration quality. a) Plot of Dice scores for all structures from 6 brains (gray lines) used in this study overlaid with the mean Dice score (black) plotted against a standard input parameter that impacts registration quality (bending energy weight). The atlas was registered to each brain using 18 different bending energy weights. **b)** Plot showing mean Euclidean distance between each of ten standard landmarks in the reference atlas and its corresponding partner in the registered brain for the identical 6 x 18 registrations used in a. Distances from Individual brains are shown in gray, mean highlighted in black. Error bars show the s.d..

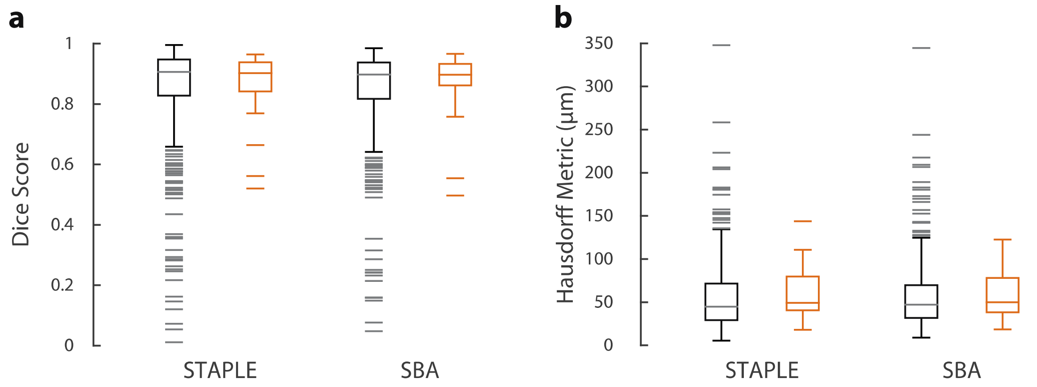

Supplementary Figure 3: Additional scoring and averaging modalities. a) Box plot showing the Dice scores of manual raters (n = 22 raters x 2 out of 4 potential brains, grey) and aMAP (orange) (n = 4 brains) for nine structures, based on the consensus-segmentation as generated by STAPLE versus SBA. There was no significant difference in median scores of human raters versus aMAP. (STAPLE: 0.91 vs 0.90, p=0.5; SBA: 0.89 vs 0.90, p=0.8; Mann-Whitney-U Test) **b)** Box plot showing the pooled Hausdorff metrics of manual raters (n = 22 raters x 2 out of 4 potential brains, grey) and the aMAP segmentations (n = 4 brains, orange) for nine structures. There was no significant difference in median scores of human raters and aMAP. (STAPLE: 45µm vs 49µm, p=0.06; SBA: 47µm vs 50µm p=0.27; Mann-Whitney-U Test).

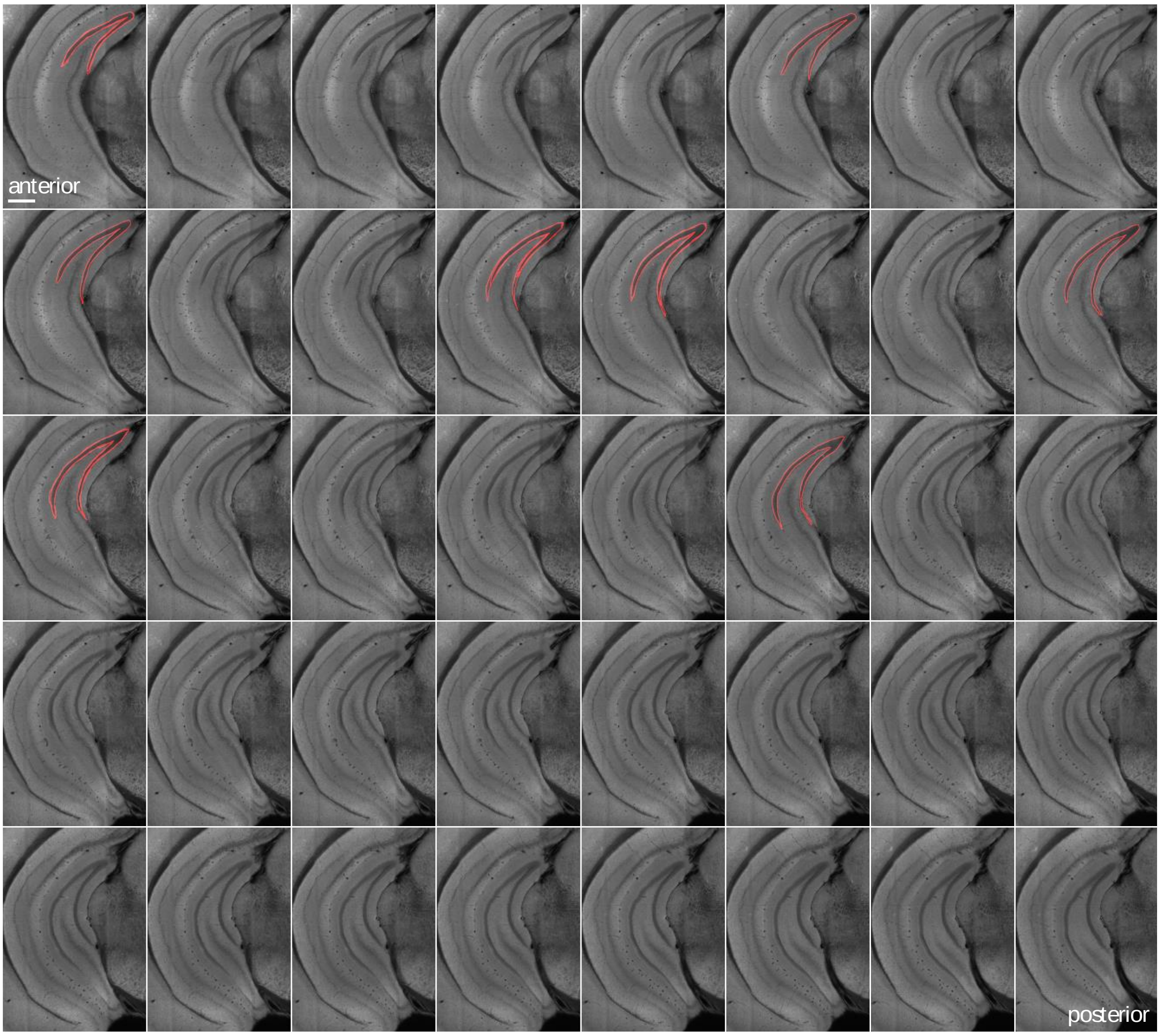

Supplementary Figure 4: Example segmentations of the DG-sg. Image series showing the area around the hippocampus on all 40 STP image sections presented to raters for the DG-sg segmentation task from one brain. Images are arranged anterior to posterior, with the raters' outlines highlighted in red. Note the distinct change in the shape of the clearly identifiable DG-sg outline from anterior to posterior. Scale bar = 500µm.

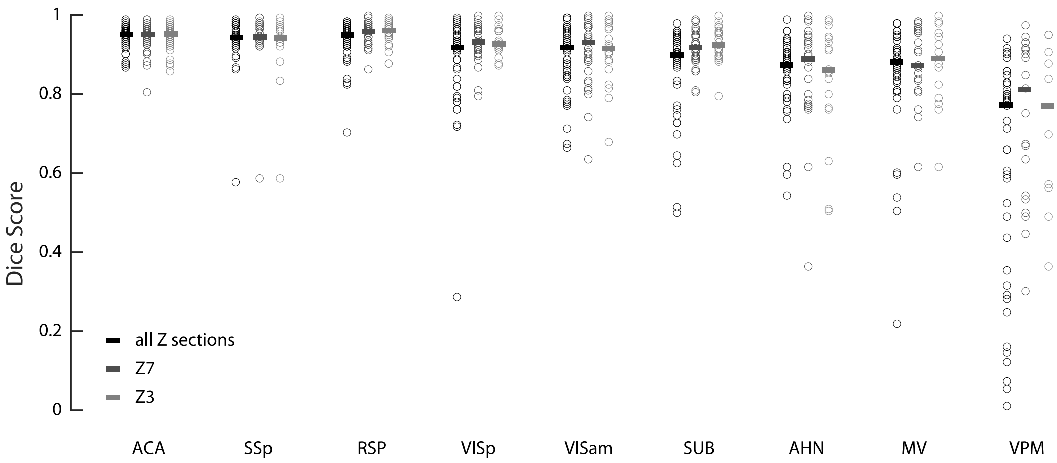

Supplementary Figure 5: Influence of Z plane on manual segmentation performance. Plot showing. Dice scores for manual segmentations performed (i) on any of the image planes, (ii) within 7 (Z7) or (iii) 3 optical sections of one another (Z3). New STAPLE consensus segmentations were calculated for each dataset. No significant differences were found between z-limited and full analyses. Median Dice scores: ACA – Full: 0.952, Z7: 0.951, Z3: 0.953; SSp – Full: 0.943, Z7: 0.944, Z3: 0.942; RSP – Full: 0.950, Z7: 0.958, Z3: 0.961; VISp – Full: 0.917, Z7: 0.932, Z3: 0.927; VISam – Full: 0.918, Z7: 0.931, Z3: 0.915; SUB – Full: 0.899, Z7: 0.918, Z3: 0.925; AHN – Full: 0.873, Z7: 0.889, Z3: 0.861; MV – Full: 0.881, Z7: 0.873, Z3: 0.890; VPM – Full: 0.772, Z7: 0.812, Z3: 0.769.

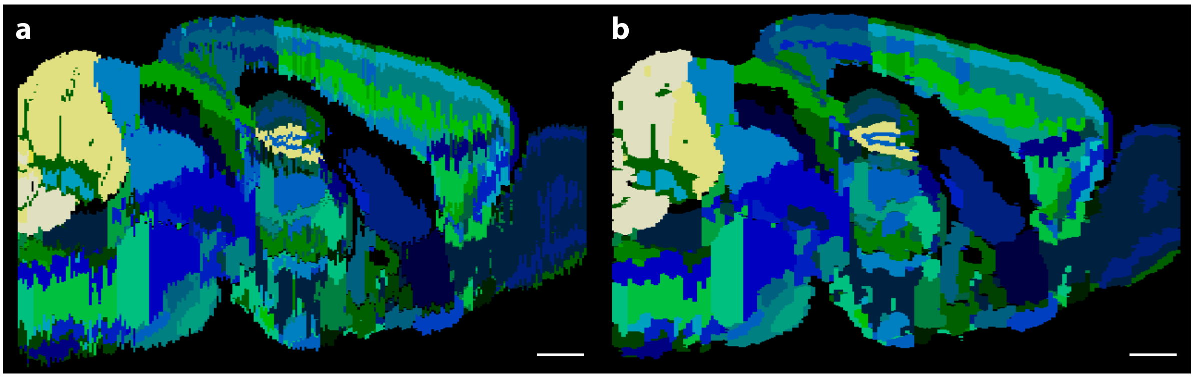

Supplementary Figure 6: Structure delineation along the anterior-posterior axis of the coronal section-based reference atlas a) Image of a sagittal section through the Kim et al. atlas (scaled to isotropic size), with the different brain areas displayed in a false color map. High frequency jitter is visible in the structure boundaries from anterior to posterior caused by artifacts stemming from the fact that the atlas is based on 2D segmentations of individual coronal cryostat sections. **b)** The same atlas section after two iterations of Gaussian smoothing of the structures, showing noticeably reduced jitter (radius 0.5 voxels at a voxel size of 20x20x50µm). Scale bars = 1mm.
